# A Digital Endocranial Cast of the Early Paleocene (Puercan) ‘Archaic’ Mammal *Onychodectes tisonensis* (Eutheria: Taeniodonta)

**DOI:** 10.1007/s10914-017-9381-1

**Published:** 2017-03-07

**Authors:** James G. Napoli, Thomas E. Williamson, Sarah L. Shelley, Stephen L. Brusatte

**Affiliations:** 1 Department of Earth, Environmental, and Planetary Sciences, Brown University, 69 Brown Street, Box 5385, Providence, Rhode Island 02912 USA; 2 School of GeoSciences, Grant Institute, University of Edinburgh, James Hutton Road, Edinburgh, EH9 3FE Scotland UK; 3 New Mexico Museum of Natural History and Science, 1801 Mountain Road, NW, Albuquerque, NM 87104-1375 USA

**Keywords:** Taeniodonta, K-Pg extinction, Paleocene, Digital endocast, Mammal radiation

## Abstract

Eutherian mammals—placentals and their closest extinct relatives—underwent a major radiation following the end-Cretaceous extinction, during which they evolved disparate anatomy and established new terrestrial ecosystems. Much about the timing, pace, and causes of this radiation remain unclear, in large part because we still know very little about the anatomy, phylogenetic relationships, and biology of the so-called ‘archaic’ eutherians that prospered during the ~10 million years after the extinction. We describe the first digital endocranial cast of a taeniodont, a bizarre group of eutherians that flourished in the early Paleogene, reconstructed from a computed tomography (CT) scan of a late Puercan (65.4 million year old) specimen of *Onychodectes tisonensis* that recovered most of the forebrain and midbrain and portions of the inner ear. Notable features of the endocast include long, broad olfactory bulbs, dorsally-positioned rhinal fissures, and a lissencephalic cerebrum. Comparison with other taxa shows that *Onychodectes* possessed some of the largest olfactory bulbs (relative to cerebral size) of any known mammal. Statistical analysis of modern mammals shows that relative olfactory bulb dimensions are not strongly correlated with body size or fossorial digging for shelter, but relative bulb width is significantly greater in taxa that habitually dig to forage for food. The anatomical description and statistical results allow us to present an ecological model for *Onychodectes* and similar taeniodonts, in which they are animals of simple behavior that rely on a strong sense of smell to locate buried food before extracting and processing it with their specialized skeletal anatomy.

**Electronic supplementary material:**

The online version of this article (doi:10.1007/s10914-017-9381-1) contains supplementary material, which is available to authorized users.

## Introduction

In the wake of the end-Cretaceous (K-Pg) mass extinction, mammals dramatically diversified, filling newly-open niche space vacated by the dinosaurs (e.g., Simpson 1949; Romer 1966; Rose 2006; Wible et al. 2007; O’Leary et al. 2013). This is an exemplary radiation in the fossil record, and it set the stage for the mammal-rich world of today. It is clear that one particular type of mammal—the eutherians, which include placentals and their closest extinct relatives—blossomed in the post-extinction world, and their dominance continues to the present. But many questions remain about the timing, pace, and drivers of the eutherian radiation (e.g., Archibald and Deutschman 2001; Goswami 2012; dos Reis et al. 2014; Grossnickle and Newham 2016). In large part, this is because we still know very little about the anatomy, phylogenetic relationships, and biology of the eutherians that prospered during the ~10 million years after the extinction, in the Paleocene (Rose 2006).

Most eutherian mammals that diversified during the early Paleocene were so-called ‘archaic’ species, which were clearly larger and more diverse than the Cretaceous eutherians but whose relationships to the modern placental orders are poorly understood (Rose 2006). Among the most familiar and distinctive of the ‘archaic’ groups are the taeniodonts, a clade of small-to-medium-sized mammals endemic to North America that originated in the Late Cretaceous, survived the extinction, and proliferated in the Paleocene (Schoch 1986; Lucas et al. 1998; Fox and Naylor 2003). Taeniodonts were among the first mammals to evolve dental adaptations for feeding on tough vegetation (e.g., crown hypsodonty) and postcranial specializations for scratch digging and/or burrowing. At least nine valid taeniodont genera are currently recognized (Schoch 1986; Rook and Hunter 2014; Williamson and Brusatte 2013). They became larger and more specialized over time (e.g., Patterson 1949), culminating in the last-surviving middle Eocene *Stylinodon*, which was about 50–100 kg in body mass and possessed rootless teeth, large gliriform canines, and powerful forelimbs with enlarged claws (Schoch 1986; Lucas et al. 1998; Turnbull 2004).

Over the past few decades, the discovery of important new fossils of taeniodonts and other ‘archaic’ mammals has helped to elucidate some aspects of their paleobiology, which in turn is starting to give insight into their evolutionary radiation (e.g., Williamson 1996). One area that has been little explored, however, is the neuroanatomy and neurosensory capabilities of these animals. The now widespread use of computed tomography (CT) scanning in paleontology has allowed for the visualization of internal cranial features and the reconstruction of brains, inner ears, and cranial vasculature, which has revolutionized the field (e.g., Witmer et al. 2008). However, few ‘archaic’ Paleocene mammals have been studied this way, so we still know very little about their brains and sense organs, how their neurosensory abilities compared to modern mammals, and how their neurobiology may have been related to their post-Cretaceous radiation.

Here, we present the first CT-based study of the braincase of a taeniodont. Using high-resolution X-ray CT, we digitally visualize and describe the endocranial cast of the basal taeniodont *Onychodectes tisonensis*. In mammals, the endocranial space is filled with the brain and other soft-tissue structures, which leave an impression on the internal surface of the surrounding skull bones (Jerison 1973; Macrini et al. 2007). An endocast, therefore, gives an indication of the general shape and volume of the brain. Because the brain is responsible for controlling and regulating the body, and plays an important role in most organismal functions, including sensory processing and analysis, motor control, and learning, studying its size and shape provides vital clues to the neurosensory abilities of this critical mammal that thrived soon after the end-Cretaceous extinction.

## Methods

### Specimen

This study is based on AMNH 785, a nearly-complete skull of *Onychodectes tisonensis* that was collected from fossil zone B of De-Na-Zin Wash of the Bisti/De-Na-Zin Wilderness Area of northwestern New Mexico, USA (Williamson 1996). This horizon represents the classic “*Polymastodon* horizon” (*Polymastodon = Taeniolabis*) of Sinclair and Granger (1914) and yields the type fauna of the late Puercan biozone of the Puercan North American Land Mammal Age (Lofgren et al. 2004). Preliminary results from ^40^Ar/^39^Ar geochronology from ash and detrital sanidine closely associated with fossil zone B indicate that it dates from within the first 500,000 years of the Paleocene (Heizler et al. 2013).

The cranium AMNH 785 was collected by a crew from the American Museum of Natural History led by Joseph Wortman in 1892, and was initially described by Osborn and Earle (1895); it was later redescribed by both Matthew (1937) and Schoch (1986). The cranium of AMNH 785 is crushed and mediolaterally distorted, and is broken in several locations. Both zygomatic arches, most of the teeth, and the occiput are missing entirely, along with a portion of the posterior skull roof. The preservation of the skull made it impossible to completely reconstruct neural anatomy, but many of the major anatomical features are visible.

**Table 1:** Comparisons of brain measurements among taxa included in comparison set. Abbreviations as follows: OBL, olfactory bulb length; OBW, olfactory bulb width; CL, cerebrum length; CW, cerebrum width; OBL/CL, olfactory bulb volume divided by cerebrum length; OBW/CW, olfactory bulb width divided by cerebrum width. Linear measurements given in mm. Body mass estimates in kg. Extinct species are not scored for forage style or mode of life. All data for extant species come from the Major National Resources for Study of Brain Anatomy website (Welker et al. 2007; http://www.brainmuseum.org) and PanTHERIA database (Jones et al. 2009) unless otherwise noted

Taxon	OBL	OBW	CL	CW	OBL/CL	OBW/CW	Forage Style	Mode	Body Mass	Reference
*Alcidedorbignya*	8.07	7.72	13.96	14.15	0.58	0.55	-	-	0.58	(Muizon et al. 2015)
*Aplodontia*	7.63	7.74	23.82	26.31	0.32	0.29	Non-digger	Fossor	0.81	-
*Capra*	16.17	25.40	55.72	53.95	0.29	0.47	Non-digger	Non-fossor	46.9	-
*Diacodexis*	8.45	15.42	20.30	26.06	0.42	0.59	-	-	0.94	(Orliac and Gilissen 2012)
*Didelphis*	13.67	11.6	20.39	20.21	0.67	0.58	Non-digger	Non-fossor	2.47	-
*Ectoganus*	18.55	25	34	30	0.55	0.83	-	-	36	(Schoch 1983, 1986)
*Equus*	33.94	28.12	114.58	91.35	0.30	0.31	Non-digger	Non-fossor	277	-
*Erinaceus*	9.48	13.19	14.39	20.47	0.66	0.64	Non-digger	Fossor	0.80	-
*Eurygenium*	15.8	22.66	52.71	57.03	0.30	0.40	-	-	119	(Dozo and Martínez 2016)
*Hyopsodus*	7.32	9.01	13.86	18.11	0.53	0.50	-	-	0.63	(Orliac et al. 2012)
*Isoodon*	11.96	12.3	18.97	22.07	0.63	0.56	Digger	Non-fossor	0.83	-
*Leptictis*	8.60	12.90	17.89	19.47	0.48	0.66	-	-	0.43	(Novacek 1982)
*Lepus*	9.37	11.66	34.97	28.25	0.27	0.41	Non-digger	Non-fossor	1.57	-
*Macropus*	12.62	10.77	36.78	28.99	0.34	0.37	Non-digger	Non-fossor	5.28	-
*Marmosa*	4.47	4.67	9	10.67	0.50	0.44	Non-digger	Non-fossor	0.05	-
*Marmota*	12.46	8.29	30.31	29.77	0.41	0.28	Non-digger	Fossor	3.88	-
*Microsyops*	8.91	11.61	21.71	26.18	0.41	0.47	-	-	1.88	-
*Mus*	5.67	4.01	9.76	11.32	0.58	0.35	Non-digger	Non-fossor	0.02	-
*Mustela*	5.48	5.97	24.17	16.87	0.23	0.36	Non-digger	Non-fossor	0.28	-
*Myrmecophaga*	26.42	33.32	59.41	50.47	0.44	0.66	Digger	Fossor	27.9	-
*Notostylops*	12	12	23	30	0.52	0.40	-	-	3.12	(Simpson 1933)
*Odocoileus*	18	19.06	55.4	44.6	0.32	0.43	Non-digger	Non-fossor	75	-
*Onychodectes*	9.5	14	18.8	16.8	0.51	0.83	-	-	4.034	Present Study
*Oryctolagus*	9.98	9.06	31.61	28.26	0.32	0.32	Non-digger	Fossor	1.59	-
*Paramys copei*	10.38	10.12	20.68	22.37	0.50	0.45	-	-	1.03	(Bertrand et al. 2016)
*Paramys delicatus*	9.94	11.46	24.5	27.17	0.41	0.42	-	-	2.70	(Bertrand et al. 2016)
*Pecari*	18.20	25.20	55.27	43.13	0.33	0.58	Digger	Non-fossor	21.3	-
*Perameles*	11.84	12.84	18.53	21.89	0.64	0.59	Digger	Non-fossor	0.72	-
*Phenacodus*	15	30	30	44.5	0.50	0.67	-	-	56	(Simpson 1933)
*Philander*	11.26	8.64	18.97	19.47	0.59	0.44	Non-digger	Non-fossor	0.43	-
*Procyon*	14.05	12.60	51.44	41.58	0.27	0.30	Non-digger	Non-fossor	6.37	-
*Rattus*	6.66	5.06	15.17	14.90	0.44	0.34	Non-digger	Fossor	0.28	-
*Rhynchippus*	14	19	52	57	0.27	0.33	-	-	85	(Dozo and Martínez 2016)
*Scalopus*	3.66	6.07	7.23	13.81	0.51	0.44	Digger	Fossor	0.09	-
*Sciurus*	9.40	9.71	29.13	26.21	0.32	0.37	Non-digger	Non-fossor	0.55	-
*Sus*	27.84	30.28	71.73	53.17	0.39	0.57	Digger	Non-fossor	84.5	-
*Tachyglossus*	15.10	19.54	34.34	38.80	0.44	0.50	Digger	Fossor	2.91	(Rismiller and McKelvey 2003)
*Tamandua*	13.79	17.68	40.88	33.24	0.34	0.53	Digger	Fossor	4.65	-
*Taxidea*	14.26	17.69	55.63	48.11	0.26	0.37	Digger	Fossor	7.84	-
*Tenrec*	4.24	3.83	7.81	12.10	0.54	0.32	Digger	Fossor	0.90	-
*Vombatus*	12.02	12.99	31.24	31.61	0.38	0.41	Non-digger	Fossor	26	-

### CT Scanning

The specimen was scanned by Dr. Hong-yu Yi with a GE phoenix v|tome|x micro-CT scanner at the American Museum of Natural History Microscopy and Imaging Facility. It was scanned with the following parameters: voltage of 170–220 kV, current of 150–260 μA, and voxel size 51.1 μm.

### CT Reconstruction

A dataset of tiff images from the CT scan was imported into Materialize Mimics 17.0 (Materialize N.V. 2014) at the University of Edinburgh School of GeoSciences. This was done to visualize and examine the internal structures of the braincase and to construct three-dimensional digital renderings of the specimen. This work was carried out by JGN.

**Fig. 1:**
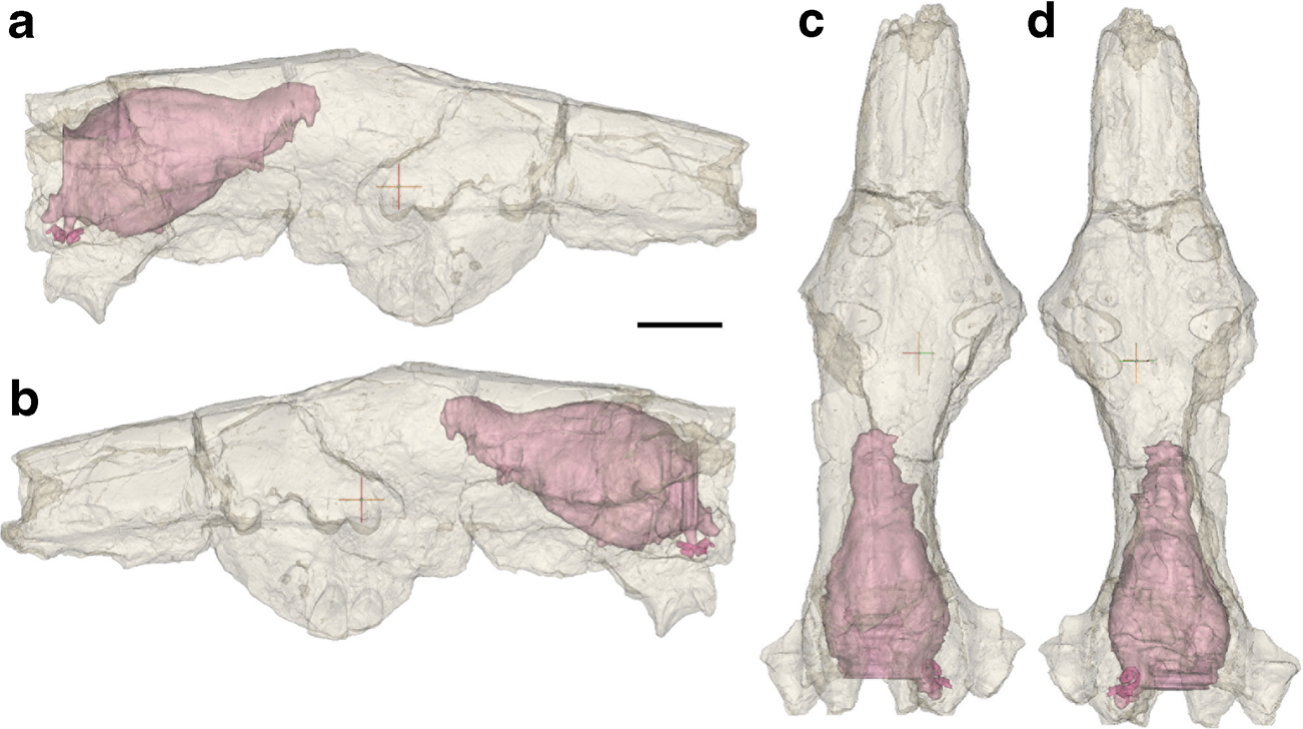
Semi-transparent digital model of AMNH 785, with the digital endocranial cast visible in its anatomical position, in: **a** right lateral view; **b** left lateral view; **c** dorsal view; **d** ventral view. Scale bar equals 10 mm

**Fig. 2:**
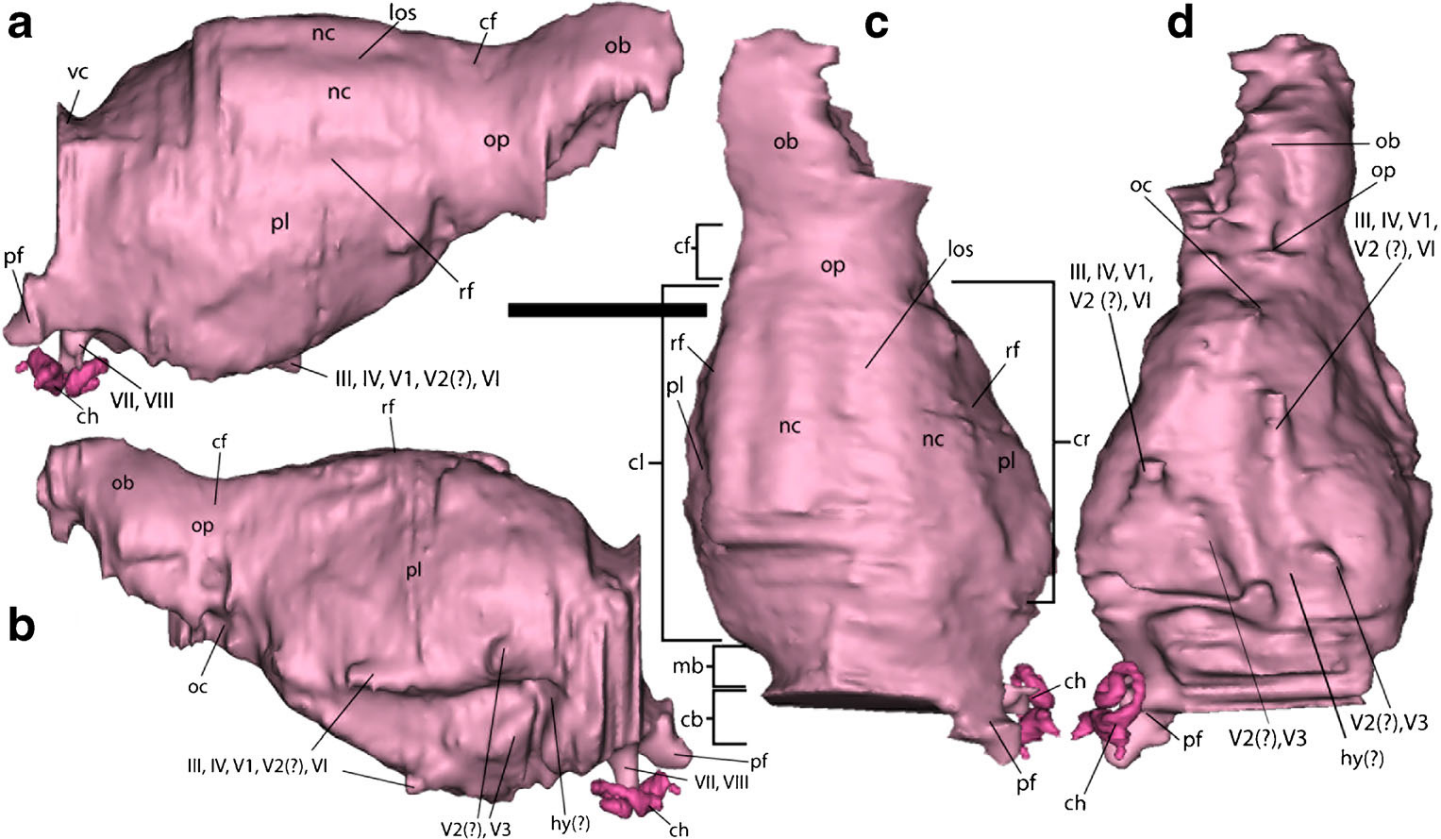
Digital endocranial cast of *Onychodectes*. Cast of cochlea is presented in a darker pink color. **a** right lateral view; **b** left lateral view; **c** dorsal view; **d** ventral view. Abbreviations are as follows: cb, cerebellum; cf., circular fissure; ch, cochlea; cl, left cerebral hemisphere; cr, right cerebral hemisphere; hy, hypophyseal fossa; los, longitudinal sulcus; mb, midbrain; nc, neocortex; ob, olfactory bulb; oc, optic chiasm; op, olfactory peduncle; pf, paraflocculus; pl; pyriform lobe; vc, vermis cerebelli; III, oculomotor nerve; IV, trochlear nerve; V_1_; ophthalmic branch of trigeminal nerve; V_2_, maxillary branch of trigeminal nerve; V_3_, mandibular branch of trigeminal nerve; VI, abducens nerve; VII, facial nerve; VIII, vestibulocochlear nerve. Scale bar equals 10 mm

The slices containing the braincase were manually segmented into several label maps. While the majority of the anterior braincase was preserved, there were many slices in which a section of it was missing, or where cracks traversed the entirety of the skull. In these cases, a straight line was drawn between preserved edges. We then used Mimics to generate surface files from the label maps, and smoothed the surfaces to reduce noise and allow proper visualization of the anatomy. The final digital endocast is presented in Figs. 1 and 2.

Due to the poor preservation of the endocranial cavity and some moderate deformation, the resulting endocast has several unnatural pits and furrows, especially on the ventral surface of the model. Thus, while the model’s outline is accurate, and most major structures are discernable, the volume of the endocast is likely substantially lower than it would have been in life. We imported the endocast model into MeshLab V1.3.3.3 (Cignoni et al. 2008) in order to calculate the volume of the endocast. We were unable to confidently measure the volume of different regions of the model (as is typical in similar analyses), and instead opted to take linear measurements to characterize the overall shape of the endocast. Such an approach is suboptimal, but less likely to be biased by the model’s irregular geometry. Future analyses using 3D volumes from better-preserved specimens may give additional insight into mammalian brain evolution.

We took orthogonal screenshots of the final model in dorsal, ventral, and left and right lateral views (Figs. 1 and 2); these screenshots were imported into Adobe Photoshop CS6 (Adobe Systems, 2012), where they were scaled to equal sizes. Linear measurements were then taken using Photoshop’s Ruler tool. We made 4 measurements of the brain; length of olfactory bulbs, width across both olfactory bulbs, length of cerebrum, and width of cerebrum. Because only the left olfactory bulb is completely preserved in AMNH 785, we had to double its width to produce a final estimate of olfactory bulb width. We first extended a line forward from the midline of the cerebrum and measured from the lateral edge of the bulb to that midline; this value was then doubled.

We calculated the encephalization quotient (EQ) of *Onychodectes*, using the equations of Jerison (1973) and Eisenberg and Wilson (1981). For these calculations, we assumed the density of brain tissue to be 0.00104 g/mm^3^ (1.04 g/cm^3^) after Barber et al. (1970). Body mass was estimated for AMNH 785 using Legendre’s (1989) mammal curve regression equation based on m1 area, whereby LogBM =1.7054(Log m1 area) + 2.2470 (BM, body mass). Estimating body mass for extinct taxa is problematic, particularly with taxa (such as taeniodonts) of contentious phylogenetic position and morphology divergent from that of extant groups. Body mass estimates based on long bone measurements are substantially more accurate than those based on dental measurements (Campione and Evans 2012). However, their use is restricted by the availability and preservation of fossil material. In the case of AMNH 785, we were restricted to the use of dental measurements, which were provided by Schoch (1986).

### Comparisons

In describing the endocast of *Onychodectes tisonensis* below, we make comparisons to several other mammal taxa, living and extinct. Our primary comparisons were with other ‘archaic’ Cenozoic eutherians: *Alcidedorbignya inopinata* (Muizon et al. 2015), *Diacodexis ilicis* (Orliac and Gilissen 2012), *Ectoganus copei* (Schoch 1983), *Eurygenium latirostris* (Dozo and Martínez 2016)*, Hyopsodus lepidus* (Orliac et al. 2012), *Leptictis dakotensis* (Novacek 1982), *Microsyops annectens* (Silcox et al. 2010), *Notostylops* sp. (Simpson 1933), *Paramys copei* and *P. delicatus* (Bertrand et al. 2016), *Phenacodus primaevus* (Simpson 1933), and *Rhynchippus equinus* (Dozo and Martínez 2016). These taxa were chosen because they are well-known representatives of major eutherian lineages phylogenetically and temporally close to *Onychodectes* (representing Paleogene members of Pantodonta, Taeniodonta, Afrotheria, Laurasiatheria, and Euarchontoglires). Digital linear measurements of olfactory bulb and cerebrum length and width of these comparative taxa were recorded from published figures with ImageJ 1.6.0 (Schneider et al. 2012) when necessary measurements were not provided (Table 1).

Further comparisons were drawn with extant mammals: *Aplodontia rufa*, *Capra hircus domestica*, *Didelphis virginiana*, *Equus burchellii*, *Erinaceus europaeus*, *Isoodon obesulus*, *Lepus americanus*, *Macropus eugenii*, *Marmosa murina*, *Marmota monax*, *Mus musculus*, *Mustela erminea*, *Myrmecophaga tridactyla*, *Odocoileus virginianus*, *Oryctolagus cuniculus*, *Pecari tajacu*, *Perameles nasuta*, *Philander opossum*, *Procyon lotor*, *Rattus norvegicus*, *Scalopus aquaticus*, *Sciurus carolinensis*, *Sus scrofa domesticus*, *Tachyglossus aculeatus*, *Tamandua tetradactyla*, *Taxidea taxus*, *Tenrec ecaudatus*, and *Vombatus ursinus*. These taxa were selected to assemble a phylogenetically broad comparative dataset that includes species with disparate modes of life, in order to better understand how brain morphology relates to ecology and phylogeny in living mammals. Images of the brains of these taxa were downloaded from the comparative mammalian brain collection of the Major National Resources for Study of Brain Anatomy website (Welker et al. 2007). Measurements of olfactory and cerebral length and width were recorded with ImageJ (Schneider et al. 2012). Estimates of average body mass for these taxa were drawn from the PanTHERIA database (Jones et al. 2009); *Tachyglossus*, a monotreme, was not included in PanTHERIA, so body mass estimates were taken from Rismiller and McKelvey (2003). Estimates of body mass for extinct taxa were taken from their respective references (Table 1).

When considering the inner ear of *Onychodectes*, comparisons were made with two key exemplar taxa for which detailed, high-resolution CT scans have been published: *Protungulatum* sp. (Orliac and O’Leary 2016) and *Leptictidium auderiense* (Ruf et al. 2016).

### Data Analysis

In addition to using the comparative taxa to help describe the endocast of *Onychodectes*, we also used their measurements to quantitatively assess the relationship between endocast proportions, body size, and ecology. All data analysis was conducted in RStudio (RStudio Team 2015), making use of the packages APE (Paradis et al. 2004), phytools (Revell 2012), picante (Kembel et al. 2010), and paleotree (Bapst 2012). A composite phylogenetic tree incorporating all comparative taxa was assembled based on Bertrand et al. (2016), Blanga-Kanfi et al. (2009), Muizon et al. (2015), Dozo and Martinez (2016), Rook and Hunter (2014), O’Leary et al. (2013), Orliac et al. (2012), and Simpson (1933). This tree is presented in ESM 2. The phylogenetic position of taeniodonts within Eutheria is unclear and currently under study by the authors; we regard their placement in this composite tree (as eutherians just outside Placentalia) as preliminary. This tree was then time-scaled, using the function ‘cal3TimePaleoPhy’ from the package paleotree, first and last appearance dates for each taxon (taken from the Paleobiology Database entries for each genus) and estimates of birth, death, and sampling rates originally calculated for Mesozoic dinosaurs (Starrfelt and Liow 2016). Branch lengths were calculated 100 times and averaged to yield a composite time-scaled tree.

Phylogenetically independent contrasts (Felsenstein 1985) for body mass, relative olfactory bulb length, and relative olfactory bulb width were calculated using the APE function ‘pic’. These contrasts were then subjected to linear regression, with body mass contrast as the independent variable and relative length or width contrast as the dependent variable (Fig. 5). The K statistic of phylogenetic signal (Blomberg et al. 2003) was calculated for the same traits using the function ‘phylosignal’ from the R package picante. Maximum likelihood ancestral state reconstruction of relative olfactory bulb length and width was calculated and mapped onto the time-calibrated phylogeny using the function ‘contMap’ from the package phytools.

*Onychodectes* and other taeniodonts exhibit skeletal features found in modern scratch diggers, such as an elongated olecranon process of the ulna and robust forelimb claws (Schoch 1986; Williamson and Brusatte 2013). However, it is difficult to determine whether this anatomy represents adaptations for foraging (for instance, habitually digging for buried foods) or other lifestyle habits such as burrowing. To assess this, we examined relative olfactory bulb size in extant mammals of various ecologies. Taxa were scored as “diggers” or “non-diggers” based on whether they habitually dig while foraging for food, and as “fossors” or “non-fossors” according to whether they habitually construct burrows. One-tailed two-sample t tests were used to determine whether extant mammals in these categories typically display significantly different relative olfactory bulb lengths and/or widths. The results are visualized in Fig. 6.

### Data Availabiliy Statement

All data required to replicate our analyses are available within the supporting information for this paper (ESM 1)﻿. These data consist of linear measurements of brain structures, ecological classifications, and first and last appearance dates for each taxon analyzed.

### Description

**Fig. 3:**
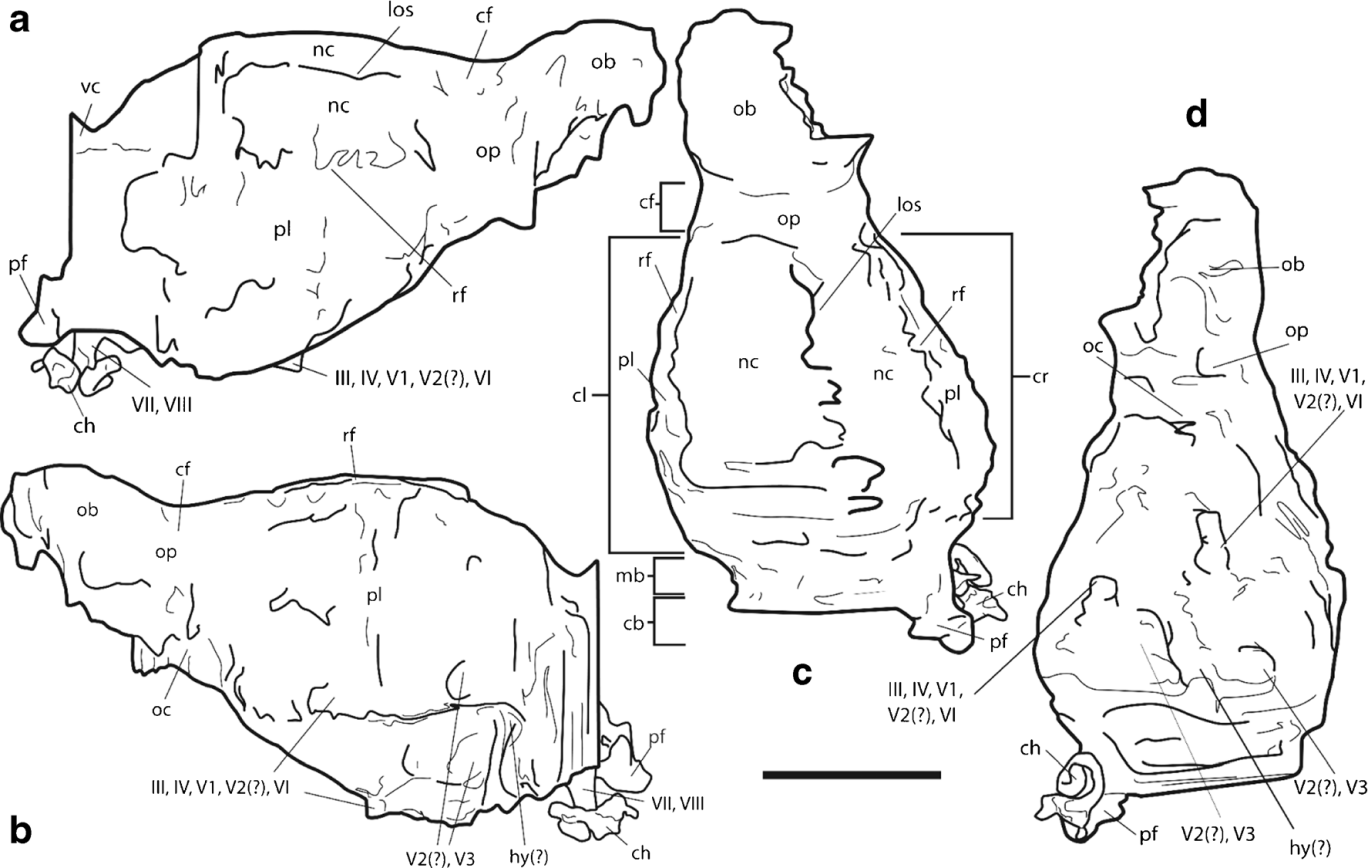
Annotated interpretive drawing of *Onychodectes* endocranial cast. **a** right lateral view; **b** left lateral view; **c** dorsal view; **d** ventral view. Abbreviations are as follows: cb, cerebellum; cf., circular fissure; ch, cochlea; cl, left cerebral hemisphere; cr, right cerebral hemisphere; hy, hypophyseal fossa; los, longitudinal sulcus; mb, midbrain; nc, neocortex; ob, olfactory bulb; oc, optic chiasm; op, olfactory peduncle; pf, paraflocculus; pl; pyriform lobe; vc, vermis cerebelli; III, oculomotor nerve; IV, trochlear nerve; V_1_, ophthalmic branch of trigeminal nerve; V_2_, maxillary branch of trigeminal nerve; V_3_, mandibular branch of trigeminal nerve; VI, abducens nerve; VII, facial nerve; VIII, vestibulocochlear nerve. Scale bar equals 10 mm

The reconstructed endocast (Figs. 1, 2, and 3) consists of the left olfactory bulb, both cerebral hemispheres, the midbrain, the anterior-most portion of the cerebellum, the paraflocculus, and portions of the inner ear (the vestibule and the cochlea). The endocast is 33.7 mm long anteroposteriorly, 16.8 mm wide mediolaterally at its widest point, and 17 mm deep dorsoventrally at its greatest depth. The brain is small relative to the cranium. It is less than half of the length of the skull, and at its widest point is narrower than the skull at the level of the orbits (Fig. 1). The dorsal portion of the endocast was reconstructed in greater detail than the ventral portion, due to matrix and bony debris in the ventral part of the braincase.

The endocast has a volume of 2467.94 mm^3^. This value, however, only reflects the volume of what parts of the endocast could be reconstructed; because the endocast lacks portions of the olfactory bulbs, cerebrum, midbrain, and hindbrain, it must be considered a minimum value. Using the minimum value of total endocast volume and a body mass estimate of 4033.85 g (from the equation of Legendre (1989) based on m1 area), we obtained an EQ for *Onychodectes* of 0.084 using the equation of Jerison (1973), and 0.10 using the equation of Eisenberg and Wilson (1981). Encephalization quotients provide a means for quantifying relative brain size as the ratio of actual brain size to expected brain size for a given body size, with 1.0 representing the average (Jerison 1973). The EQ of *Onychodectes* is substantially lower than that calculated for other ‘archaic’ taxa such as *Alcidedorbignya* (0.42) (Muizon et al. 2015), *Hyopsodus* (0.36–0.49) (Orliac et al. 2012), *Pantolambda* (0.33) (Silcox et al. 2010), and *Phenacodus* (0.20) (Silcox et al. 2010). The closest taxon, in terms of EQ, to *Onychodectes* is the pantodont *Barylambda*, which has an EQ of 0.11 using the Jerison (1973) equation or 0.10 using the Eisenberg and Wilson (1981) equation (Silcox et al. 2010).

It is important to recognize that our EQ estimates are only an approximation due to the poor preservation of the specimen and limited choice of body mass estimators. However, increasing the brain volume by 25% and 50% does little to alter the resulting EQ, producing values of 0.11 and 0.13, respectively (using the Jerison (1973) equation). Thus, it is apparent that whatever its exact value, the EQ of *Onychodectes* was very low.

### Telencephalon

#### Olfactory Bulbs

The olfactory bulbs (Figs. 2 and 3) are poorly preserved. The left bulb is almost fully reconstructed, although partially crushed, and only the posterior portion of the right bulb is present. The complete left bulb is longer anteroposteriorly (9.5 mm) than wide mediolaterally (6.4 mm). It is ovoid in shape and widens posteriorly, and it does not pass between the orbits. Portions of the right bulb attach to the medial margin of the left bulb along its length, indicating that the two bulbs were at least partially conjoined in life. The overall poor preservation of the olfactory bulbs makes it impossible to accurately reconstruct their volume, so we cannot make any confident assertions about the ratio of olfactory bulb mass to body mass in *Onychodectes*. The ratio of the length of the olfactory bulb to the length of the cerebrum is 0.51, and the ratio of the width of the olfactory bulb to the width of the cerebrum is 0.74.

Among our comparison taxa, the olfactory bulbs of *Onychodectes* are relatively longer than those of *Diacodexis, Eurygenium*, *Leptictis*, *Microsyops*, *Paramys*, and *Rhynchippus*, and are relatively shorter than those of *Alcidedorbignya*, *Ectoganus*, *Hyopsodus*, and *Phenacodus*. Only *Ectoganus* has olfactory bulbs relatively wider than those of *Onychodectes*.

The left bulb is well separated from the cerebrum, which does not overlap it (Figs. 2 and 3). This reflects the condition seen in all of the other extinct comparison taxa. The olfactory peduncles of *Onychodectes* are transversely broad, with a poorly developed circular fissure separating them from the cerebrum; therefore, the olfactory bulbs and cerebrum have a gradual (rather than distinct) division (Figs. 2 and 3). This condition is similar to that seen in *Alcidedorbignya* and *Ectoganus,* and stands in contrast to the clearly-demarcated boundary between olfactory bulb and cerebrum in all other extinct comparison taxa. While the cribiform plate is not preserved, the ~45° incline of the ventral surface of the olfactory bulbs suggests that the bulbs contacted the plate at a similar angle.

#### Cerebrum

The cerebrum (Figs. 2 and 3) is reconstructed almost entirely, but the dorsal portion of the posterior cerebrum could not be reconstructed because the skull roof is broken in that area. It is 18.8 mm long and 16.8 mm wide, and is narrowest at its anterior end. The cerebrum widens posteriorly, such that the cerebrum’s most lateral extent is more laterally positioned than the tip of the paraflocculus. Its depth is relatively constant along its length, so it appears roughly circular in lateral view. The highest point of the cerebrum occurs near its posterior limit, and is ventral to the dorsalmost point of the olfactory bulbs. The cerebrum appears to have lacked gyri and sulci, making it lissencephalic. *Alcidedorbignya*, *Ectoganus*, *Hyopsodus*, *Microsyops*, and *Rhyphodon* also have lissencephalic cerebra, in contrast to the gyrencephalic *Diacodexis*, *Eurygenium*, *Notostylops*, *Paramys copei*, *Paramys delicatus*, *Phenacodus*, and *Rhynchippus*.

The two cerebral hemispheres are clearly divided along their length by a broad, shallow longitudinal sulcus (Figs. 2 and 3). The posterior end of each hemisphere is discernable, but the transverse sulci are not readily distinguishable. In life, these sulci housed venous sinuses; the longitudinal sulcus would have housed the superior sagittal sinus, which would have divided posteriorly and travelled laterally as two transverse sinuses within the transverse sulcus.

The rhinal fissures develop at the boundary between the dorsal and lateral pallia (Liem et al. 2001); in adult mammals, the rhinal fissures demarcate the pyriform lobe (a derivative of the lateral pallium) and the remaining parts of the telencephalon (most notably the neocortex) (Miller 1964; Liem et al. 2001). There is some confusion about this terminology in the literature. Some prior studies have referred to what we are calling the pyriform lobe as the “paleocortex.” However, as it is most commonly used, “paleocortex” is a type of cortex that occurs on the medial and inferior portion of the temporal lobe. Thus, not only does ‘paleocortex’ (in this usage) not refer to a particular structure, but the structure referred to as “paleocortex” (what we are calling the pyriform lobe) is in fact comprised of archicortex (Purves et al. 2001). We therefore recommend referring to this structure as the pyriform lobe. If the anatomy of the sub-rhinal fissure area is too poorly resolved to allow confident identification of the pyriform lobe, it is best to refer to it as the rhinencephalon, which is an anatomical region including the pyriform lobe, olfactory bulbs, amygdala, and hippocampus, among other structures (Miller 1964). In primates and rodents, the rhinal fissure’s position on an endocast is marked by the raised cast of the overlying orbitotemporal canal (Silcox et al. 2010; Bertrand et al. 2016). Given that the rhinal fissures on the *Onychodectes* endocast (Figs. 2 and 3) appear as depressions, not ridges, it seems that the orbitotemporal canal did not follow the path of the rhinal fissures in this taxon.

In *Onychodectes*, the rhinal fissures are clearly visible on both hemispheres of the cerebrum; they are positioned high on the cerebrum and are visible in dorsal view (Figs. 2 and 3). The neocortex of *Onychodectes* is therefore inferred to have been restricted to the dorsal aspect of the cerebrum; a much larger pyriform lobe comprised the ventral aspect. The neocortex maintains a roughly constant width along its length, and does not widen posteriorly. The pyriform lobe, in contrast, widens and deepens posteriorly, giving it a subtriangular outline in lateral view (Figs. 2 and 3). As the cerebrum is overall little expanded (see below), it is most likely that the apparent large size of the pyriform lobe is due to the small size of the neocortex.

The rhinal fissures of *Alcidedorbignya*, *Ectoganus*, and *Onychodectes* are relatively high on the endocast, suggesting that they all had small neocortices. None of the three show a posterior widening of the neocortex, in contrast with *Diacodexis*, *Eurygenium*, *Hyopsodus*, *Microsyops*, *Notostylops*, *Paramys*, *Phenacodus*, and *Rhynchippus*. The neocortex of *Onychodectes* is small relative to the expanded neocortex typical of more derived eutherians, such as *Diacodexis*, *Eurygenium*, *Hyopsodus*, *Microsyops*, *Notostylops*, *Paramys*, *Phenacodus*, and *Rhynchippus*, but similar in size to that of *Alcidedorbignya* and *Ectoganus*.

#### Mesencephalon

The tectum of the midbrain is broadly exposed in dorsal view, with a maximum anteroposterior exposure distance of 5.3 mm (Figs. 2 and 3). Its exposure is 28% of the length of the cerebrum. Several species (including *Pleuraspidotherium*, *Artocyon*, and *Artocyonides*) have been reported to show longer exposure of the midbrain (Orliac et al. 2012). Among the comparison taxa in this study, *Alcidedorbignya*, *Diacodexis*, *Ectoganus*, *Hyopsodus*, *Phenacodus*, and possibly *Leptictis* also show long exposure of the midbrain. *Microsyops*, *Paramys copei*, *Paramys delicatus*, and *Rhyphodon*, on the other hand, have a more restricted dorsal midbrain exposure.

Overall, the midbrain is broadly exposed and the cerebrum little expanded. The midbrain is sloped posteroventrally (Figs. 2 and 3). This region of the brain is relatively poorly preserved, so finer details are difficult to accurately discern. Neither inferior nor superior colliculi are visible on the endocast. It is possible that their absence is due to the overall poor preservation of the region; alternatively, the colliculi could have been completely covered by soft tissues, rendering them absent on the endocast.

In *Alcidedorbignya*, *Ectoganus*, and *Phenacodus*, the cerebrum is small relative to the size of the cranium, as is the case in *Onychodectes*, while the other taxa have fairly enlarged cerebra. Midbrain exposure in *Alicdedorbignya*, *Ectoganus*, *Onychodectes*, and *Phenacodus* is therefore likely an artifact of their small cerebra, and as such probably does not reflect any adaptive difference in the brain. As no colliculi were reconstructed for *Onychodectes*, they cannot be compared to those of any of the comparison taxa.

#### Cerebellum

Only the anteriormost portion of the cerebellum (Figs. 2 and 3) was reconstructed, due to the poor preservation of the posterior portion of the cranium. The anteriormost portion of the cerebellum is horizontal; what is preserved is positioned lower than the entirety of the cerebrum. The anterior part of the vermis cerebelli is likely visible, but the endocast is not detailed or complete enough to clearly demarcate its boundaries. The right paraflocculus is also reconstructed as a 4 mm long lobe projecting posteroventrolaterally from the cerebellum (Figs. 2 and 3). The shape of the paraflocculus is irregular, appearing rounded in some views and subconical in others. It is large in relation to the brain, at approximately 40% of the length of the olfactory bulbs.

#### Cranial Nerves

It was not possible to reconstruct the optic chiasm in detail because of matrix and bony fragments in its vicinity. However, vestiges of the optic nerves are present at the anterior end of the ventral aspect of the cerebrum (Figs 2 and 3). The traces of cranial nerves III-VI (oculomotor, trochlear, trigeminal, and abducens) were able to be reconstructed on the ventral surface of the endocast, and the most proximal portion of nerves VII and VIII (facial and vestibulocochlear) are visible exiting the brain and contacting the cochlea (Figs. 2 and 3). Nerves III, IV, V_1_, and VI emerge from the ventral aspect of the endocast midway along its length and travel anteriorly together. Posterior to these, a separate nerve bud (likely V_3_) exits the brain and travels anteriorly. The location of V_2_ is equivocal; it is possible that its specific exit from the brain was obscured by poor preservation, but more likely is that it is conjoined with the traces of III, IV, V_1_, and VI, as in other ‘archaic’ species (e.g., Orliac et al. 2012; Dozo and Martinez 2016; Bertrand et al. 2016).

Due to the poor preservation of the specimen, cranial nerve foramina on the skull are indistinct. However, drawing on previous studies of ‘archaic’ mammals (e.g., Orliac et al. 2012; Dozo and Martinez 2016; Bertrand et al. 2016), III, IV, V_1_, and VI probably exited through the sphenorbital fissure. V_2_ exited through the foramen rotundum or sphenorbital fissure, and V_3_ through the foramen ovale or sphenotympanic fissure. VII and VIII exited through the internal acoustic meatus. The location of the hypophyseal fossa is difficult to discern; it seems to be located at the posterior end of the cerebrum, at approximately the level the exit of V_3_ from the brain.

#### Inner Ear

**Fig. 4:**
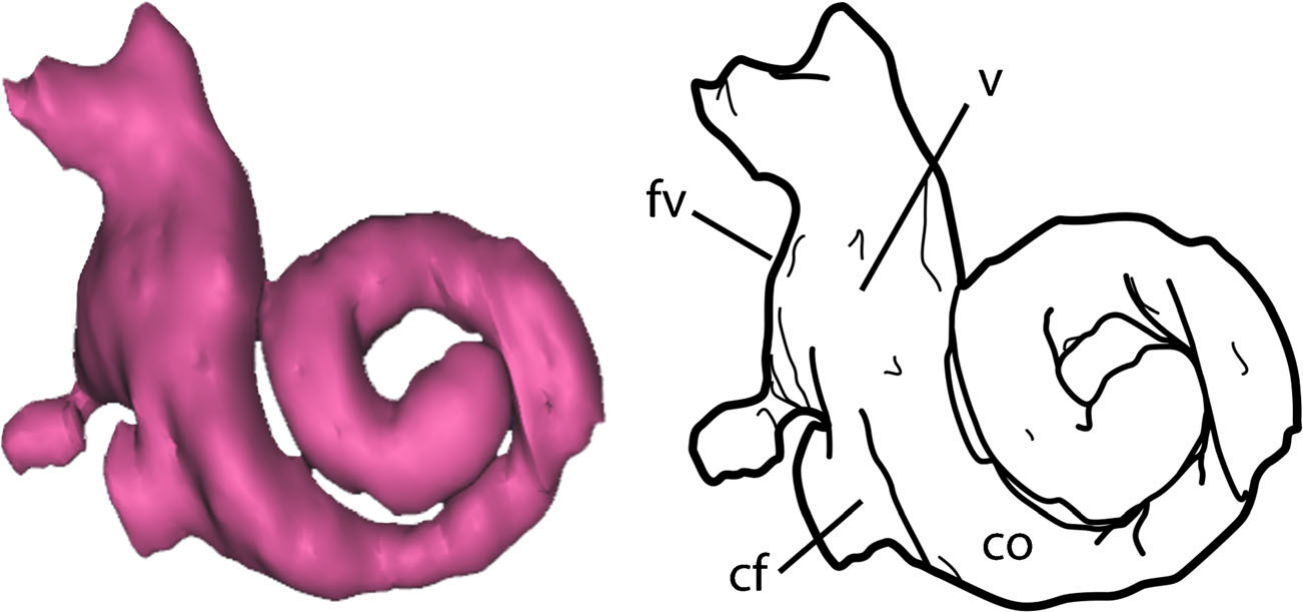
Annotated interpretive drawing of the inner ear of *Onychodectes*. Abbreviations are as follows: cf, cochlear fossula; co; cochlear canal; fv, fenestra vestibuli (line pointing behind edge of cochlea because the opening is not quite visible in this view); v, vestibule. Scale bar =1 mm

The inner ear of the right side of the head was partially reconstructed (Figs. 2, 3, and 4). The cochlea and vestibule are complete. Unfortunately, the delicate semicircular canals are damaged and could not be reconstructed.

The cochlea is aligned anteroposteriorly; the cochlear canals are anterior (and slightly lateral) to the vestibule. The vestibule is dorsoventrally high (3.2 mm high) but anteroposteriorly short (1.8 mm wide), giving it a tall appearance in lateral view. The entire cochlea is aligned in a roughly parasagittal plane, so the coils of the cochlea canal and full height of the vestibule are visible in ventrolateral view. This contrasts with the condition in *Protungulatum*, in which the cochlea is oriented in a roughly transverse plane, so the spiral of the cochlear canals is best visible in dorsal or ventral view (Orliac and O’Leary 2016). However, in both taxa the long axis of the cochlea extends anteroposteriorly, such that the cochlear canals are anterior to the vestibule. *Leptictidium*, in contrast, has cochlear canals that are medial and slightly ventral to the vestibule, so the long axis of the cochlea projects medioventrally (Ruf et al. 2016).

These differences in the overall orientation of the cochlea in the three taxa cause the major landmarks of the cochlea (such as the fenestra vestibuli) to face in different directions. In *Onychodectes*, the fenestra vestibuli is directed posterodorsally, while the aperture of the cochlear fossula opens posteroventrally. In *Protungulatum*, the fenestra vestibuli points ventrally and the aperture of the cochlear fossula points medially. Finally, in *Leptictidium*, the fenestra vestibuli points posterolaterally and the aperture of the cochlear fossula points laterally.

The cochlear canal of *Onychodectes* is 1.8 mm long. The successive turns of the cochlear canal are well separated, while in *Protungulatum* and *Leptictidium* successive turns of the cochlear canal contact one another. In *Onychodectes*, the canal makes approximately 1.25 turns, while in *Protungulatum* it makes 1.54 and in *Leptictidium* it makes 2.25 (Orliac and O’Leary 2016; Ruf et al. 2016). The diameter of the cochlear canals of *Onychodectes* is roughly constant along its length. The canals of *Onychodectes* are similar in relative diameter to those of *Protungulatum*, but relatively more gracile than the thick canals seen in *Leptictidium*. The cochlea is 2 mm high and 3 mm wide; the cochlear aspect ratio is 0.66 (calculated according to Ekdale (2013) whereby the height of the spiral is divided by the width of the basal turn). A high cochlear aspect ratio is considered to be above 0.55. Thus, the cochlear aspect ratio of *Onychodectes* is considered high and is greater than that seen in *Protungulatum* (0.51) and *Leptictidium* (0.65) (Orliac and O’Leary 2016; Ruf et al. 2016).

#### Statistics

**Fig. 5:**
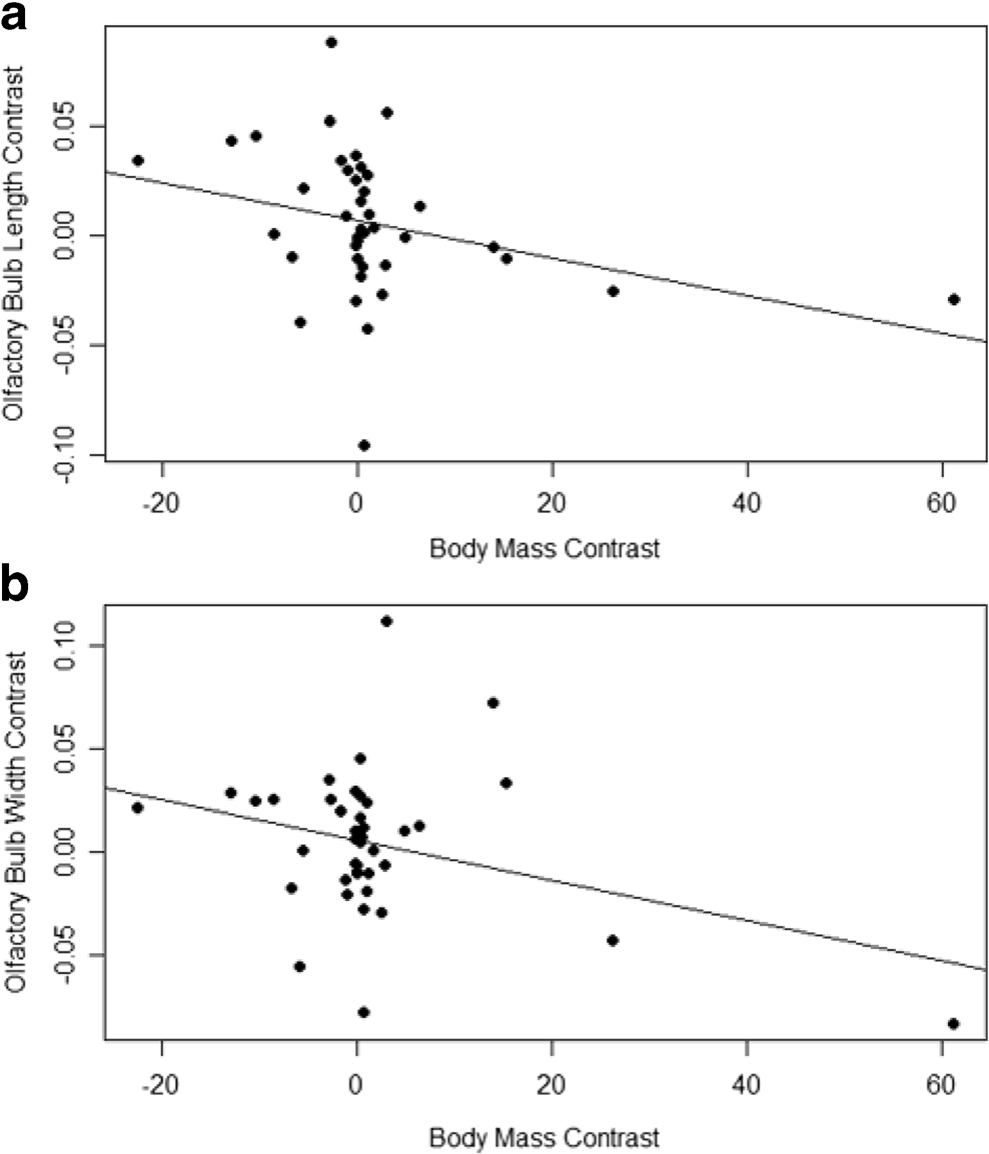
Phylogenetic regressions of olfactory bulb dimensions and body mass. **a** olfactory bulb width and body mass (*p* = 0.08, R^2^ = 0.0565, b = −0.0009).; **b** olfactory bulb length and body mass (*p* < 0.05, R^2^ = 0.07932, b = −0.0009). Body mass exerts little effect on the dimensions of the olfactory bulb, so dimensions can be compared among taxa of varying size

Phylogenetically-corrected regressions (Fig. 4) demonstrate that both relative olfactory bulb length and width are not strongly related to body mass among mammals in our dataset. Relative length (Fig. 5a) was found to have a weak but significant negative relationship with body size (*p* < 0.05, R^2^ = 0.07932, b = −0.0009), while relative width (Fig. 5b) has a weak and insignificant relationship (*p* = 0.08, R^2^ = 0.0565, b = −0.0009).

**Fig. 6:**
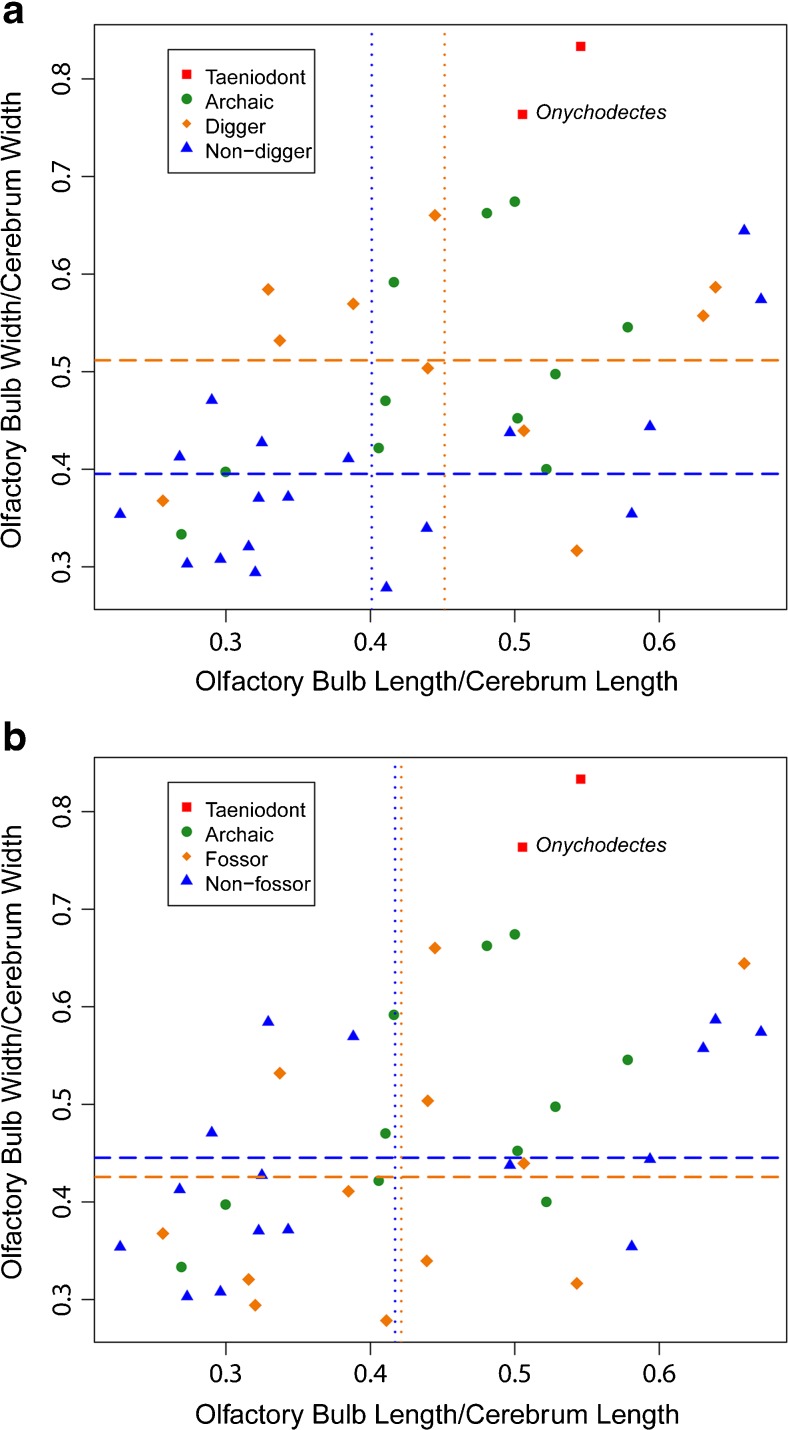
**a** Relative olfactory bulb length plotted against relative olfactory bulb width for all taxa in the comparison dataset. Dotted lines denote mean values of relative length for diggers (orange) and non-diggers (blue). Dashed lines denote mean values of relative width for diggers (orange) and non-diggers (blue). Among difference of mean length between diggers and non-diggers was insignificant (*p* = 0.1786), while the difference in mean width was significant (*p* < 0.01); **b** Relative olfactory bulb length plotted against relative olfactory bulb width for all taxa in the comparison dataset. Dotted lines denote mean values of relative length for fossors (orange) and non-fossors (blue). Dashed lines denote mean values of relative width for fossors (orange) and non-fossors (blue). Neither the difference in mean length or width between the two categories was significant (*p* = 0.46 and *p* = 0.67, respectively)

One-tailed two-sample t tests (Fig. 6) show that relative olfactory bulb width is significantly greater in digging vs. non-digging animals (*p* < 0.01), while relative olfactory bulb length is not (*p* = 0.1786). Neither relative olfactory bulb length nor width exhibits significant correlation with fossorial or non-fossorial behavior (*p* = 0.46 and *p* = 0.67, respectively).

**Fig. 7:**
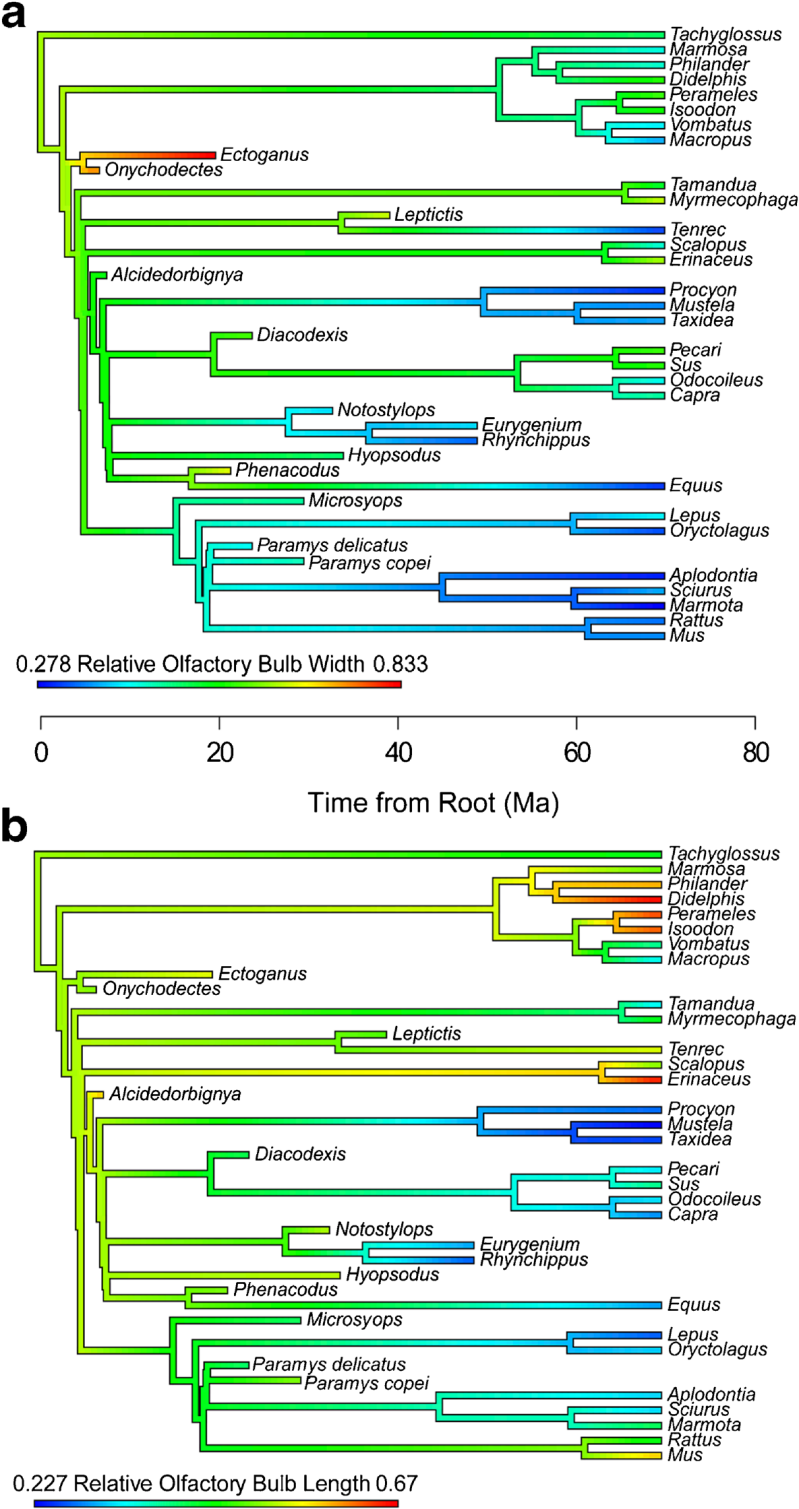
Composite phylogenetic tree of all taxa in comparison dataset, with branches colored according to character state values for **a** relative olfactory bulb width and **b** relative olfactory bulb length. Warmer colors indicate larger values

Relative olfactory bulb width exhibits a strong phylogenetic signal with K > 1 (*p* < 0.05), indicating that closely related species resemble each other more than would be expected if the trait’s evolution were random. The K statistic for relative bulb length is less than 1 (*p* < 0.05), indicating a weak phylogenetic relationship and thus that taxa are more different than would be expected by chance. These results are corroborated by ancestral state reconstruction (Fig. 7) showing that relative olfactory bulb width is fairly stable within clades, but that relative olfactory bulb length is more variable.

## Discussion

### Comparison to *Ectoganus copei*

The only taeniodont for which an endocranial cast has been described is *Ectoganus copei*, a large, robust, highly specialized Eocene species that was one of the last surviving taeniodonts. A partial endocast of this taxon was described by Schoch (1983) based on a cast (made from a RTV-silicone rubber compound) of skull USNM 12714, a specimen first described by Gazin (1936) and later designated as the holotype of *Ectoganus copei* by Schoch (1981). This skull retains a portion of the deciduous dentition and thus represents an immature individual, which may make comparisons with other mammals problematic because we still know very little about how taeniodonts (and most other ‘archaic’ mammals) changed during ontogeny. Furthermore, this skull is missing the ventral portion of the braincase, meaning that only a partial endocast could be reconstructed and described.

The anterior portion of the olfactory bulbs of USNM 12714 is missing, so their length relative to the cerebrum cannot be easily compared to that of *Onychodectes*. However, Schoch (1983) drew estimated bounds for the olfactory bulbs of *Ectoganus* 3.4 mm in front of their anterior extent; if these bounds are accurate, the bulbs are equal in relative length to those of *Onychodectes.* The ratio of the width of the olfactory bulbs of USNM 12714 to its cerebral width is 0.83, making its bulbs relatively wider than those of *Onychodectes* (ratio = 0.76); thus, *Ectoganus* seems to have had olfactory bulbs slightly larger than those of its close relative. The bulbs of *Onychodectes* appear to be more elongate in shape than those of *Ectoganus*, but this is not certain because of the missing anterior portions of the bulbs in USNM 12714. The two taeniodonts have similarly broad olfactory peduncles, which are not overlain by the cerebrum.

Both *Onychodectes* and *Ectoganus* possess lissencephalic cerebra, with rhinal fissures occurring fairly high on the cerebrum and visible in dorsal view. The rhinal fissures remain parallel to the midline along their lengths. Therefore, both taxa possessed fairly small neocortices that were limited to the dorsal portion of the cerebrum. Schoch (1983) stated that the pyriform lobe and neocortex of *Ectoganus* were subequal in size; this stands in contrast to the condition in *Onychodectes*, in which the neocortex was definitively smaller than the pyriform lobe. However, it is worth noting that the different preservation of *Ectoganus* or its juvenile status could have affected the apparent size of its neocortex.

In both taxa, the tectum of the midbrain is exposed in dorsal view. The length of exposure of *Onychodectes* is ~23% of the length of the cerebrum, similar to that found in *Ectoganus* (~20%). The area is not reconstructed in detail in either endocast, so the features of the midbrain (e.g., the colliculi) cannot be compared between taxa. The inner ear and paraflocculus of *Ectoganus* were not reconstructed, and so cannot be compared with those of *Onychodectes*.

Overall, the digital *Onychodectes* endocast adds significant new information to our knowledge of taeniodont neural anatomy. This specimen provides for the first time the cranial nerve stems and a complete olfactory bulb for a taeniodont. Additionally, this specimen provides us with the first glimpses of the inner ear and paraflocculus for a taeniodont, neither of which was recovered in Schoch’s (1983) physical peel of USNM 12714.

### Comparisons to Extant Mammals

We here summarize the most salient comparisons between *Onychodectes* and modern mammals, which help give insight into the neurosensory anatomy and behaviors of this extinct taeniodont.

At a gross level, the virtual endocast of *Onychodectes* appears most similar to the brain of the Virginia opossum (*Didelphis virginiana*). Both have large olfactory bulbs relative to the size of their cerebra (width ratio = 0.57 for *Didelphis* and 0.76 for *Onychodectes*) with broad olfactory peduncles, and both have midbrains with dorsal exposure. *Didelphis*, however, has a much larger neocortex that widens posteriorly, and some faint convolution of the neocortex.

Notably, *Onychodectes* has remarkably large olfactory bulbs. While some modern mammals in our dataset (*Didelphis*, *Erinaceus*, *Isoodon*, *Mus*, *Perameles*, *Philander*, *Scalopus*, and *Tenrec*) possess olfactory bulbs relatively longer than those of *Onychodectes* and *Ectoganus*, none have olfactory bulbs as relatively wide. Extant comparison taxa show a range of olfactory bulb width to cerebral width ratios of 0.28–0.66 (with the hedgehog *Erinaceus* and the anteater *Myrmecophaga* at the high end of the spectrum), while *Onychodectes* has a ratio of 0.76. *Onychodectes* therefore has some of the proportionally largest olfactory bulbs yet known in Mammalia, with only its close relative *Ectoganus* having larger ones among the taxa we have studied.

*Onychodectes* additionally has a neocortex that is restricted to the dorsal part of the endocast. The only extant mammals in our dataset with neocortices so dorsally restricted as those of *Onychodectes* are the European hedgehog (*Erinaceus europaeus*), the tail-less tenrec (*Tenrec ecaudatus*), and the southern brown bandicoot (*Isoodon obesulus*). In all three taxa, the neocortex is restricted to the dorsal aspect of the cerebrum and the rhinal fissure and pyriform lobe are visible in dorsal view. In *Tenrec*, the neocortex does not widen posteriorly; this is similar to the condition in *Onychodectes* but contrasts with that seen in *Erinaceus* and *Isoodon*. Additionally, the elongate morphology of the brain of *Onychodectes* is distinct from the short, deep brain of *Tenrec*. In all other modern mammals in the comparison dataset, the neocortex is expanded such that the rhinal fissure and pyriform lobe are no longer visible in dorsal view. In many placentals, the neocortex is so greatly expanded that the rhinal fissures and pyriform lobe are hidden from dorsal and lateral views almost entirely.

Neocortical folding is absent in *Onychodectes* and seems exceptionally phylogenetically variable among extinct and modern taxa. The brain of *Tachyglossus* has well-developed gyri and sulci. Among marsupials, bandicoots (*Isoodon* and *Perameles*) and opossums (*Didelphis*, *Marmosa*, and *Philander*) have only faint convolution, while *Macropus* and *Vombatus* (especially the latter) show well-developed gyri and sulci. Convolution is also variable in placentals. *Capra, Equus*, *Myrmecophaga*, *Odocoileus, Pecari, Sus*, and *Tamandua* all exhibit well-developed gyri and sulci. *Erinaceus*, *Lepus, Marmota, Oryctolagus*, and *Sciurus* display structures on the cerebrum that may represent very weakly developed gyri and sulci, while *Mus*, *Tenrec*, and *Rattus* have completely lissencephalic cerebra. Among extinct mammals, *Alcidedorbignya*, *Ectoganus*, and *Leptictis* are lissencephalic, while *Diacodexis*, *Eurygenium*, *Microsyops*, *Notostylops*, *Paramys*, *Phenacodus*, and *Rhynchippus* are gyrencephalic. Lissencephalic taxa appear on various branches of the mammal family tree, implying that the evolution of neocortical folding was fairly complex, with several acquisitions and reversals; further research may show how many times it was acquired and lost within mammals.

*Onychodectes* exhibits a dorsally-exposed midbrain, which is also found in *Didelphis*, *Erinaceus*, *Isoodon*, *Perameles*, and *Philander* – a set of animals with life histories and behaviors dissimilar to each other and to *Onychodectes*, but which all possess neocortices of limited expansion. *Alcidedorbignya*, *Ectoganus*, and *Phenacodus* also have dorsally-exposed midbrains and relatively small cerebra, and differ enough skeletally that they likely held disparate lifestyles. Given that no obvious behaviors correlate to dorsal midbrain exposure in these species, we consider it probable that the dorsal exposure of the midbrain of *Onychodectes* is a result of the small size of its cerebrum, not a specific adaptation.

### Paleobiology

While the endocast of *Onychodectes* is admittedly incomplete, its morphology yields valuable insight into the sensory abilities and behavior of both *Onychodectes* and taeniodonts in general.

The most striking feature of this endocast is its relatively large, elongate olfactory bulb, which ranks among the largest olfactory bulbs known from any ‘archaic’ eutherian. Phylogenetic regressions (Fig. 5) demonstrate that both relative olfactory bulb length and width are not strongly related to body mass among mammals in our dataset: relative length was found to have a weak but significant negative relationship with body size, while relative width has a weak and insignificant relationship. Therefore, olfactory bulb dimensions show little correlation to body size, and instead likely reflect their organism’s ecology. Olfactory bulb size is correlated, at least partially, with olfactory acuity (Bhatnagar and Kallen 1974). Thus, it follows that the exaggerated size of this structure implies that *Onychodectes* had a relatively keen sense of smell.

This hypothesis is reinforced by the presence of a large pyriform lobe. In modern mammals (and presumably *Onychodectes*), the rhinal fissure demarcates the boundary between the neocortex and the pyriform lobe (Bertrand et al. 2016), which functions as the main olfactory cortex (Liem et al. 2001). While we cannot directly determine the size of the olfactory cortex of *Onychodectes*, the large pyriform lobe suggests that it could have had a large region of its brain dedicated to processing olfactory inputs.

The high olfactory acuity of *Onychodectes* is probably related to a suite of other unusual features of this animal. *Onychodectes* and other taeniodonts possess high-crowned molars, large temporal fossae (which provide space for enormous jaw adductors), and postcranial adaptations for scratch-digging (Schoch 1986). These features indicate that *Onychodectes* habitually dug for buried, tough foods such as roots and tubers. An acute sense of smell would be expected in such an animal, as keen olfaction would enable it to more effectively locate buried food. We suggest that *Onychodectes* probably located food by smell before digging it out and processing it with its powerful jaws and robust teeth. Modern pigs (*Sus scrofa*), which habitually dig for tough food, are like *Onychodectes* in possessing massive, elongate olfactory bulbs (Schmidt 2014), lending support to this behavioral hypothesis.

Relative olfactory bulb dimensions are associated with certain ecological habits in modern mammals (Fig. 6). Relative olfactory bulb width is significantly greater in digging vs. non-digging animals (Fig. 6a), while no statistically discernable difference exists for relative length (*p* = 0.1786). Therefore, relative olfactory bulb width is likely the better predictor of foraging behavior among these species. Neither relative olfactory bulb length nor width exhibits significant correlation with fossorial or non-fossorial behavior (Fig. 6b), so we argue that it is digging in the context of foraging that most likely drives the evolution of large olfactory bulbs.

The inference that olfactory bulb width is related to ecology is further supported by Blomberg’s K statistic (Blomberg et al. 2003). Relative olfactory bulb width exhibits a strong phylogenetic signal with K > 1, indicating that closely related species resemble each other more than would be expected if the trait’s evolution were random. We interpret this as indicating that relative olfactory bulb width is fairly stable within clades whose members employ similar feeding strategies (e.g., among Carnivora and Rodentia). This finding is depicted graphically in Fig. 7a, which shows that closely related pairs of “diggers” (such as *Tamandua* and *Myrmecophaga*) show similar values for relative olfactory bulb width. The K statistic for relative bulb length is less than 1, indicating a weak phylogenetic relationship and thus that taxa are more different than would be expected by chance. This trait tends to be highly variable, even within clades of similar ecology (Fig. 7b). Relative olfactory bulb length is likely being driven by a factor other than chance (otherwise the K statistic would be indistinguishable from 1), but foraging habit doesn’t seem to be the driver. Future research will help clarify the roles of many potential drivers of mammal brain morphology, but the most important result of our analyses is that relative olfactory bulb width is strongly tied to foraging ecology in modern mammals.

The relation of relative olfactory bulb width to foraging habit has implications for our understanding of taeniodonts. Figs. 6 and 7 demonstrate that *Onychodectes* and *Ectoganus* have extremely wide olfactory bulbs in comparison to other taxa, which strongly supports the hypothesis that they were specialized scratch-digging foragers. Schoch (1986) suggested that *Onychodectes* may have been partially arboreal, based on observations of postcranial features that are likely retained from a smaller, more arboreal ancestor. Our results cast doubt on this assessment, and mesh nicely with Williamson and Brusatte’s (2013) findings that catalogued postcranial adaptations for scratch-digging in most taeniodonts, including *Onychodectes*. The brain anatomy of *Onychodectes* does not rule out the possibility of burrowing, which is certainly a plausible strategy for the animal (the burrowing American badger *Taxidea taxus* is of a similar size and has a broadly similar skeleton), but there is little affirmative evidence from its brain anatomy to assert that *Onychodectes* was a habitual burrower. Such evidence would most likely have to come from other portions of the anatomy, but this is outside of the scope of this paper.

Another aspect of the *Onychodectes* endocast with possible biological relevance is its dorsally-positioned neocortex. We were unable to quantify this character for statistical comparison to other taxa due to several factors, including its three-dimensional morphology and the extreme neocortical expansion in many placentals, which often envelops the pyriform lobe and renders it invisible in external view. However, the neocortex of *Onychodectes* is qualitatively distinct from that of other taxa. In this taeniodont, the neocortex is restricted to the medial part of the dorsal aspect of the cerebrum, making it proportionally small relative to the remainder of the brain. This notably contrasts with the expansive neocortex of many more derived placentals (Liem et al. 2001), and also with the more derived taeniodont *Ectoganus* (Schoch 1983). The neocortex of modern mammals has a variety of functions, including sensory integration (primarily visual and auditory), and facilitating cognitive processes of learning, memory, communication, and reasoning (Liem et al. 2001; Rakic 2009). In modern mammals, increased neocortical size is associated with the development of numerous higher cognitive functions and behaviors (Rakic 2009; Orliac and Gilissen 2012). The small neocortex of *Onychodectes* implies that the animal’s higher cognitive functions (such as learning and memory) and behavior would appear simplistic alongside modern eutherians. The similarly-sized neocortices of *Onychodectes* and *Ectoganus* suggest that this was the case for all taeniodonts; however, more research on the clade’s endocranial anatomy is required before confirming this. It is important to note, however, that taeniodonts were fairly successful in their ecosystems (in terms of their diversity and abundance), so while probably simplistic in intelligence by modern standards they were likely well-equipped for their own time and place.

Neocortical folding is theorized to be a mechanism of increasing the size of the neocortex while fitting it into a relatively smaller braincase (Kelava et al. 2013). Because the neocortex of *Oncychodectes* is already small, the lissencephaly of its cerebrum is perhaps unsurprising. The adaptive significance of lissencephaly or gyrencephaly remains unclear but is presumably related to the development of higher cognitive functions associated with neocortical expansion (Rakic 2009; Kelava et al. 2013).

Given the inferred limited cognitive abilities of *Onychodectes*, it is likely that the ancestral taeniodont was an animal of fairly simple behavior, at least relative to most modern placentals. ‘Archaic’ eutherians in our dataset – specifically *Alcidedorbignya*, *Ectoganus*, *Hyopsodus*, *Leptictis*, *Onychodectes*, and *Phenacodus* – exhibit dorsally-restricted neocortices and small cerebra. The presence of this condition in ‘archaic’ eutherians suggests that these animals would have been capable of cognition at a level similar to that of *Onychodectes*, and small neocortices and cerebra may be plesiomorphic for Eutheria. If these taxa are representative of the ancestral eutherian and/or placental (that is, if their brains represent the plesiomorphic state for Eutheria/Placentalia and not phenotypic reversals), then it could be argued that the initial radiation of eutherians after the end-Cretaceous extinction had little to do with innovations in neurosensory anatomy and behavior.

Endocasts of Cretaceous mammals are equivocal about the ancestral condition for the clade; some taxa have small neocortices, others large ones, while some lack rhinal fissures entirely (which, while not precluding the presence of a neocortex, offers no help in assessing its size) (Kielan-Jaworowska and Trofimov 1986; Macrini et al. 2007). Future research will undoubtedly clarify these ancestral conditions and refine our understanding of neocortical evolution. However, it is worth noting that the brain of *Onychodectes* was grossly similar to that of Cretaceous theriiform *Vincelestes* (Macrini et al. 2007), and is therefore potentially representative of the ancestral eutherian.

In sum, it seems that the ancestral eutherian was fairly simplistic in both cognition and behavior. This is not to say that these animals were unspecialized; clearly, basal eutherians acquired neurological adaptations for their respective lifestyles, as evidenced by the vastly enlarged olfactory bulbs of *Onychodectes* and *Ectoganus*. However, the anatomical structures associated with the high-level cognition typical of modern placentals seem to have evolved after the initial radiation of Eutheria/Placentalia, and therefore would not have given early eutherians a competitive edge over other taxa during their radiation. Rather, such cognition appears to have arisen well after the group had risen to ecological dominance.

Instead, in light of recent work on the diets and cranial disparity of Cretaceous eutherians (Grossnickle and Newham 2016), we suggest that the development of new craniodental features linked to a greater diversity of diets was a prime driver of the initial Cretaceous radiation of this clade. Then, after the end-Cretaceous extinction wiped out a large number of mammals, the surviving eutherians diversified into newly vacant ecospace (Archibald 2011; Wilson 2013). The number of species and their anatomical disparity greatly increased (Halliday et al. 2016; Longrich et al. 2016), maximum body sizes became much larger (Alroy 1999; Slater 2013), and many new diets and locomotor behaviors appeared (Rose 2006). Only afterwards did more derived eutherians, seemingly those more closely related to the modern placental orders, develop the signature neurological toolkit of today’s big-brained, highly-cognitive mammals.

## Conclusions

The brain of *Onychodectes* is generally similar to those of other ‘archaic’ eutherians, such as its close relative *Ectoganus* and the pandodont *Alcidedorbignya*, and may be representative of the ancestral eutherian condition. The main distinguishing feature of its brain is its exaggerated olfactory bulbs, which are among the largest known from any eutherian mammal (with only *Ectoganus* having larger ones among the taxa we considered), suggesting that *Onychodectes* had an acute sense of smell. However, the animal’s relatively small neocortex indicates that it lacked the capacity for high-level cognition and social behaviors common in modern eutherians. Analysis of how olfactory bulb size relates to ecology in modern mammals, coupled with observations of the skeletal anatomy of *Onychodectes*, reveals that this ‘archaic’ mammal was likely a scratch-digging forager that habitually dug for tough, buried food such as roots and tubers.

## Institutional Abbreviations

AMNH, American Museum of Natural History (New York); USNM, National Museum of Natural History (Washington, D.C.)

## Electronic supplementary material

ESM 1 (XLSX 20 kb)

ESM 2 (PDF 160 kb)
