# Supplementary material for: A Digital Endocranial Cast of the Early Paleocene (Puercan) ‘Archaic’ Mammal Onychodectes tisonensis (Eutheria: Taeniodonta)
Source: J Mamm Evol. 2017 Mar 7;25(2):179–95. doi: 10.1007/s10914-017-9381-1 (PMC5938319; doi:10.1007/s10914-017-9381-1)

A Digital Endocranial Cast of the Early Paleocene (Puercan) 'Archaic' Mammal *Onychodectes tisonensis* (Eutheria: Taeniodonta)

James G. Napoli[1,2], Thomas E. Williamson[3], Sarah L. Shelley[2], Stephen L. Brusatte[2,3]

[1]Department of Earth, Environmental, and Planetary Sciences, Brown University, 69 Brown Street, Box 5385, Providence, Rhode Island 02912.

*Corresponding Author. Email: james_napoli@brown.edu Phone: (631) 680-8973

[2]School of GeoSciences, University of Edinburgh, Grant Institute, James Hutton Road, Edinburgh EH9 3FE, Scotland, UK.

[3]New Mexico Museum of Natural History and Science, 1801 Mountain Road, NW, Albuquerque, NM 87104-1375, USA

Figures on next two pages.

Fig. 7 Composite phylogenetic tree assembled based on Bertrand et al. (2016), Blanga-Kanfi et al. (2009), de Muizon et al. (2015), Dozo and Martinez (2015), Rook and Hunter (2013), O'Leary et al. (2013), Orliac et al. (2012), and Simpson (1933).

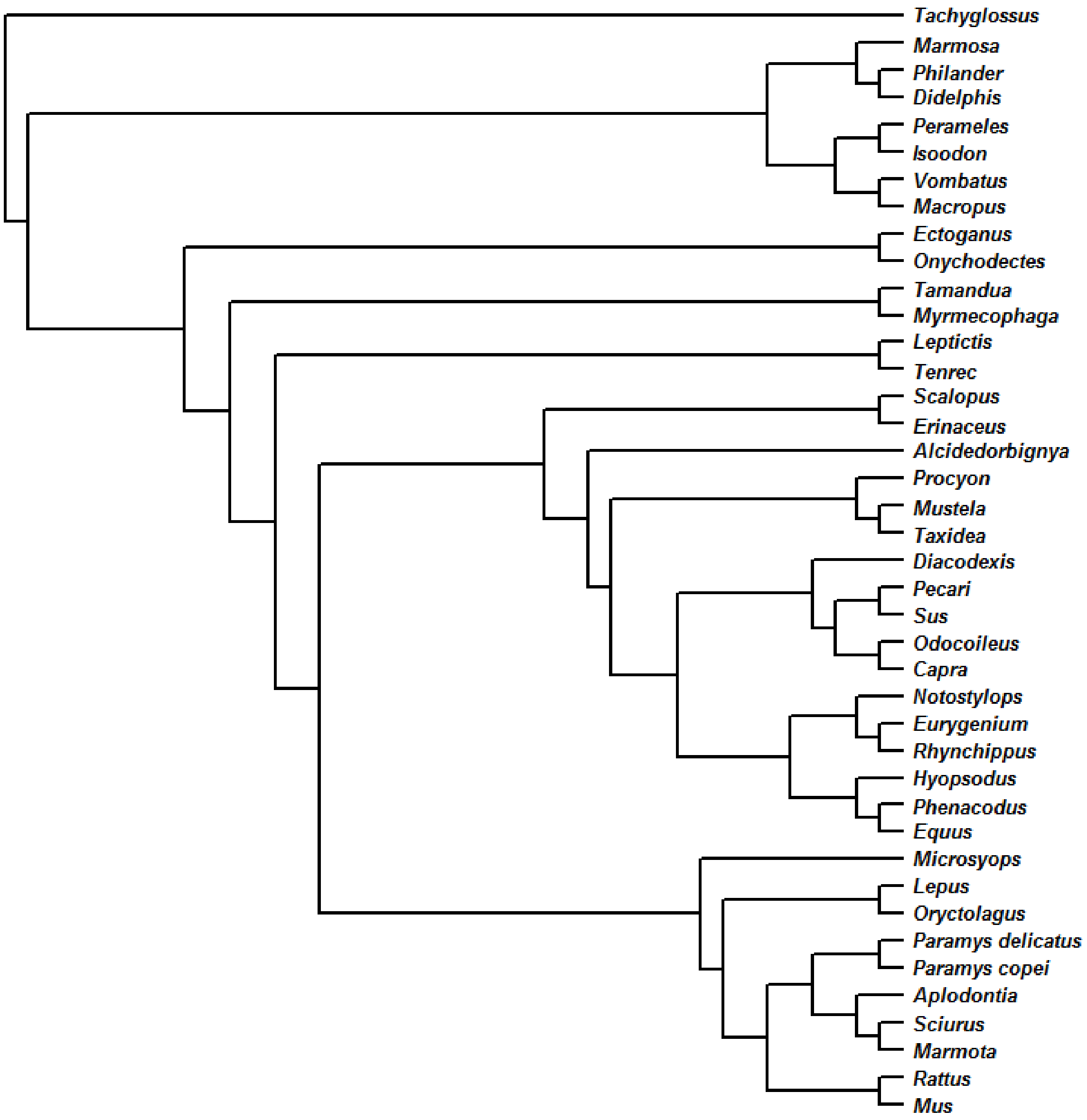

Fig. 8 Same phylogenetic tree as in Fig. 7, after branch-length estimation and time scaling.

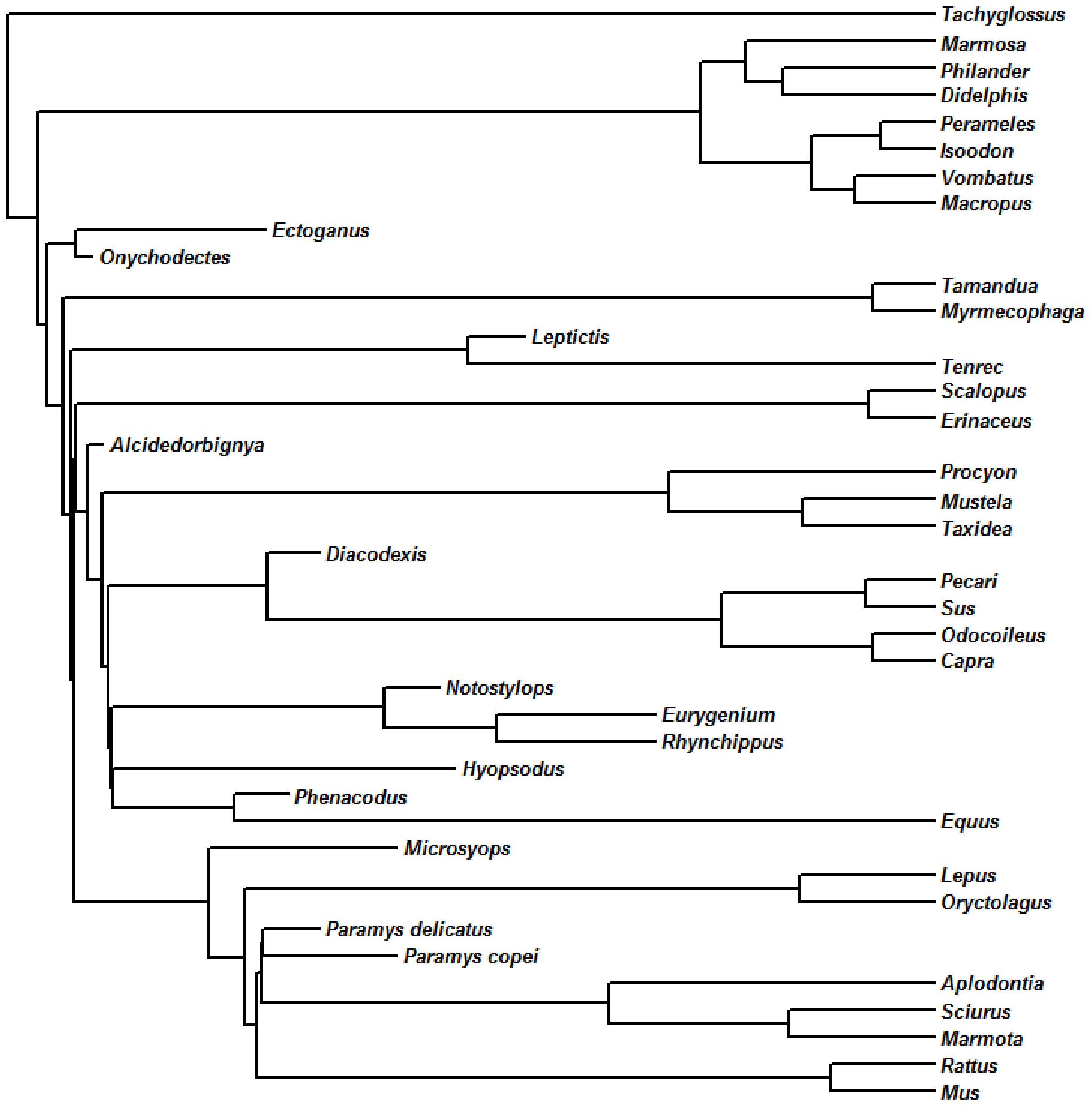

Supplement: Supplementary file 2 — (PDF 160 kb) [file 10914_2017_9381_MOESM2_ESM.pdf]
